# Biohybrid Nanoparticle-Based In Situ Monitoring of In Vivo Drug Delivery

**DOI:** 10.3390/bios13121017

**Published:** 2023-12-06

**Authors:** Sohee Ju, Hyeon-Yeol Cho

**Affiliations:** Department of Bio & Fermentation Convergence Technology, Kookmin University, 77 Jeongneung-ro, Seongbuk-gu, Seoul 02707, Republic of Korea; wnthgml97@kookmin.ac.kr

**Keywords:** biohybrid nanoparticle, in situ drug release, real-time monitoring

## Abstract

Nanomaterials have gained huge attention worldwide owing to their unique physicochemical characteristics which enable their applications in the field of biomedicine and drug delivery systems. Although nanodrug delivery systems (NDDSs) have better target specificity and bioavailability than traditional drug delivery systems, their behavior and clearance mechanisms in living subjects remain unclear. In this regard, the importance of bioimaging methods has come to the forefront for investigating the biodistribution of nanocarriers and discovering drug release mechanisms in vivo. In this review, we introduce several examples of biohybrid nanoparticles and their clinical applications, focusing on their advantages and limitations. The various bioimaging methods for monitoring the fate of nanodrugs in biological systems and the future perspectives of NDDSs have also been discussed.

## 1. Introduction

Nanoparticles are 1–100 nm in size and have great potential for biomedical usage [1]. With the increasing importance of improving the accuracy of early diagnosis and drug delivery, several nanomedicine disciplines have been developed by fusing nanotechnology and medical technology [2]. Nanotechnology has been rapidly developed and has contributed to enhanced drug delivery systems by employing nanoscale materials in diagnosis, therapy, and bioimaging [3,4,5,6]. Owing to their small size and huge surface area, nanoparticles can encapsulate therapeutic agents and penetrate cell membranes, enabling more efficient and controlled drug delivery in vivo than conventional drug delivery systems [7]. With the development of nanodrug delivery systems (NDDSs), the in vivo behavior of drug-loaded nanoparticles has been intensively investigated in recent years. Since nanoparticles are not nature-generated materials, considering their effects on living organisms is essential. Their drug delivery mechanism, accumulation, and elimination in vivo need to be examined for toxicological study and further study [8,9,10]. To study the biodistribution of nanocarriers, numerous bioimaging techniques have been developed [11,12,13,14,15]; these techniques allow non-invasive real-time monitoring of nanocarriers in vitro and in vivo, enabling the fate tracking of nanodrugs and investigation of their degeneration procedures and clearance mechanisms [16,17].

Here, we introduce several examples of biohybrid nanoparticles and their clinical applications with commonly used monitoring methods such as fluorescence-based bioimaging techniques, magnetic resonance imaging (MRI), and surface-enhanced Raman spectroscopy (SERS) to discover the behavior of nanoparticles in vivo. Additionally, the importance of nanoparticle fate tracking and degeneration procedures will be addressed for future investigation.

## 2. Design of Biohybrid Nanoparticles for Drug Delivery

Nanoparticles are materials with distinctive physical and chemical properties [1] and have created a huge sensation in many areas. Particularly, nanoparticles are widely used in imaging, diagnosis, and treatment due to their biosimilarity, huge surface area, and ease of control over particle characteristics [4,7,18,19]. Consequently, nanomedicines are gaining traction in commercialization and achieving remarkable success compared to traditional medical approaches [20].

The development of nanoscience and nanotechnology has opened up new possibilities for boosting or enabling whole new functionalities in biological systems [2,3]. The convergence of materials engineering and biological science created nanohybrids by combining functional nanomaterials with living systems [21]. Nano-biohybrids employ artificial nanomaterials to give organisms emergent traits that are outside the range of their evolutionary potential. Consequently, they confer new or increased intrinsic or exogenous features such as enhanced stress tolerance, regulated metabolism and proliferation, artificial photosynthesis, or conductivity [22,23]. The synthetic component can consist of inorganic materials [24,25,26], organic materials, or hybrid materials [27], while the biological component can range from simple biomolecules such as DNA and proteins to complex biological systems such as living cells, tissues, or organisms. The selection of both biological and synthetic components affects the ultimate functionality of biohybrid systems. In this review, we discuss the different nanoparticles used as therapeutic agents for drug delivery and for in vivo and in vitro monitoring after conjugation with biological substances (Table 1).

### 2.1. Nucleic Acid-Based Nanoparticles (NANPs)

Being building blocks of life, DNA and RNA are involved in data storage and acquisition. NANPs can be constructed in the desired size, form, and composition using the laws of base pairing [28]. Moreover, aptamer, which is a short oligonucleotide that can bind target molecules, is widely used in biosensing and drug delivery due to its target specificity and ease of functionalization [29,30]. Considering their capacity to build themselves and recognize desired target, nucleic acids are ideal candidates for structuring nanoparticles. Remarkably, gene treatments paired with nanotechnology have expanded the therapeutic and biological uses of NANPs, including biosensing, gene silencing, protein replacement, and vaccination [31].

**Figure 1:**
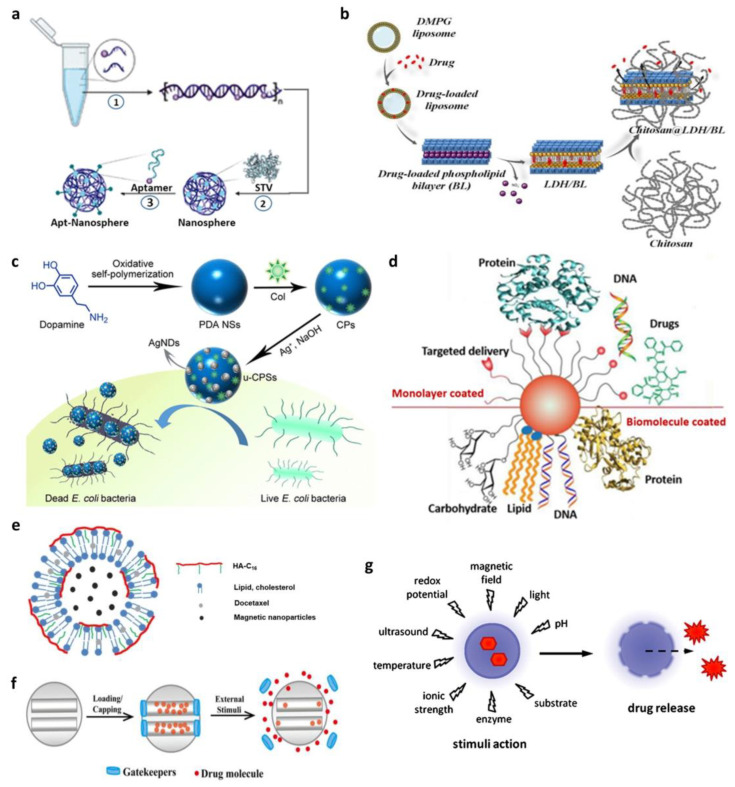
Different schematic representations of biohybrid nanoparticles for in vivo and in vitro applications. (**a**) Illustration of the three steps of aptamer-conjugated hybrid nanosphere self-assembly. (Adapted with permission from Ref. [32]. Copyright 2022 American Chemical Society.) (**b**) Sequential steps to develop biohybrid drug delivery system. (Reprinted from Ref. [35].) (**c**) Schematic illustration of the synthesis of silver-based nanomaterials and their antibacterial application. (Reprinted with permission from Ref. [36]. Copyright 2020 American Chemical Society.) (**d**) Schematic presentation of the AuNP surface-coating materials employed in delivery systems. (Reprinted with permission from Ref. [37]. Copyright 2012 Elsevier.) (**e**) Schematic representation of the preparation of nanohybrid magnetic liposomes encapsulating an anticancer drug. (Adapted with permission from Ref. [38]. Copyright 2017 Elsevier.) (**f**) Schematic representation of gatekeepers on the pore outlets of mesoporous silica nanoparticles (MSNs) for stimuli-responsive controlled drug delivery systems (CDDSs). (Adapted with permission from Ref. [39]. Copyright 2015 Elsevier.) (**g**) Schematic illustration of stimuli drug-release reaction of drug-loaded nanocarrier. (Reprinted with permission from Ref. [40]. Copyright 2012 Elsevier.).

Recently, Song et al. created hybrid nanospheres using the two most essential macromolecules—DNA and proteins [32]. They created the nanosphere through a simple one-pot assembly system (Figure 1a). The tetrameric protein streptavidin helped four biotinylated DNA molecules to be bound, resulting in a DNA–protein co-assembled nanostructure. The synthesized nanosphere could easily be loaded with chemotherapeutic agents and had great target specificity due to functionalization with aptamers. This proved the wide application range of NANPs as well as their high efficiency and versatility as nanodrug carriers for advanced targeted therapies.

As mentioned above, nucleic acids have such unique features like target specificity, great biocompatibility, and a self-building ability, making them attractive candidates for drug carriers. However, they are intrinsically less stable than inorganic substrates, leading to failure of drug delivery and limitations of their clinical applications. Many researchers have been investigating the improvement in nucleic acid-based nanomaterials’ stability in physiological conditions recently. For example, Lee et al. used histone to manipulate the mechanical property of developed DNA microscaffold and increase cell retention time [33]. In their study, they fabricated a therapeutic matrix by assembling a reforming cellular DNA hydrogel (rCDH) with mammalian cells as building blocks. This ultrasoft matrix was injected into wounded skin, and as cells proliferated, rCDH gradually degenerated and cells could be dislodged. To successfully introduce the therapeutic cells to damaged tissue, DNA should be disintegrated slowly, and histone was the key. In another study, Wu et al. developed DNA nanowires (NWs) for anticancer drug delivery that can last at least 24 h in serum condition [34]. The NWs were constructed with six single-stranded DNA including aptamers which target cancer cells by hiding all nicks from nuclease attack. The NWs had enhanced nuclease degeneration resistance and increased drug-loading capacity due to the π-π stacking of DNAs.

### 2.2. Liposomes

With a particle size of 400 nm, liposomes are concentric bilayer vesicles consisting of either manufactured or naturally occurring phospholipids [41]. Phospholipid molecules consist of a polar phosphate group and two hydrophobic fatty acid chains and spontaneously self-assemble into a closed, bilayer structure in an aqueous environment. The hydrophilic phosphate groups are exposed to the external environment and also form the inner layer that surrounds the aqueous core of the liposome, while the hydrophobic fatty chains are stacked and packed in between the two hydrophilic layers. Therefore, liposomes are used to transport substrates that are lipophilic or water-soluble in the lipid bilayer cavity, performing intracellular and site-specific drug delivery with therapeutic effectiveness and safety [42].

Drug delivery systems meet certain needs, such as lowering toxicity, increasing the efficiency of drug loading and release, or improving the biodistribution in various organs, all of which are rarely met with two-component nanoparticles. Tichit et al. constructed a unique biohybrid drug delivery system by intercalating an Mg/Al-NO_3_-layered double hydroxide (LDH) with ibuprofenate anions (IBU) or a phospholipid bilayer (BL) with a neutral medication, such as 17-estradiol, and then embedding it in chitosan beads (Figure 1b) [35].

Liposomes often arrive at their site of action by extravagating from the circulation into the interstitial space [43]. Liposomes can be also utilized as target selectors and their activity against extracellular diseases can be improved. Both passive and active techniques can be used for targeting liposomes to particular tissues. This is due to the ease with which liposomes may be altered by including extra molecules on the lipid bilayer’s outer surface. However, liposomes are often quickly eliminated because of their size and high degradability. The generation of therapeutically effective liposomes is so hampered by opsonization that they have been addressed by almost every research documented in the literature. One of the ways to decrease liposome opsonization to prevent clearance and lengthen their circulation half-life is PEGylation [44]. Therefore, choosing valid materials to increase the durability of liposomes in vivo is necessary in further investigations.

### 2.3. Metallic Nanoparticles

Metal nanoparticles have engineered sizes and structures that facilitate their usage as biomedicine carriers, catalysts, and imaging reagents [26]. Moreover, the base of the metal nanoparticles imparts additional unique features, such as magnetic properties [45]. Since they are created bottom-up from atoms, metal nanoparticles are frequently employed in nano-application sectors. Metallic nanoparticles including silver, gold, and zinc nanoparticles have tunable optical characteristics and a huge capacity for surface functionalization. However, their clinical applications are sometimes limited owing to toxicity concerns [46,47,48]. Therefore, when developing metallic nanoparticles, examination of their biocompatibility and their modification using methods such as surface coating are crucial to avoid possible immunological rejection.

#### 2.3.1. Silver Nanoparticles (AgNPs)

AgNPs have sparked attention in the field of biomedicine owing to their antibacterial and anticancer properties [49]. The Ag(0) state of silver may not change in vitro but in vivo, it is quickly etched by body electrolyte components and turned into positive ions, which are extremely harmful to cell membranes. Other biological actions of AgNPs, including bone mending and wound repair, vaccine immunogenicity enhancement, and antidiabetic properties, have also been investigated [50]. Understanding the biological mechanisms and possible cytotoxicity of AgNPs will allow more effective medical applications.

Wu et al. developed powerful antibacterial nanospheres (NSs) decorated with silver nanodots (Figure 1c) [36]. The NSs had a polydopamine (PDA) surface functionalized with an antibiotic (colistin) and decorated uniformly with tiny silver nanodots. It showed synergistic bactericidal efficiency in treating bacterial infections. PDA NSs might adhere to bacterial surfaces as an adhesive nanocarrier, allowing medicines on the PDA surfaces to be released persistently via a near-infrared laser-triggered mechanism. Furthermore, the PDA surface can be loaded with other antibiotics for wider clinical applications. Similarly, a novel material for wound dressing composed of chitosan-L-glutamic acid (l-GA)–hyaluronic acid (HA) solution loaded with AgNPs was fabricated in a recent study [51]. The synthesized sponge-like material promoted wound healing, combining three substrates while resisting bacterial infection with AgNPs. In another study, Li and Qiu developed a unique drug delivery system using AgNPs covered with camptothecin (CPT)-based polymer prodrugs [52]. Due to the nanoparticle surface energy transfer (NSET) effect, the fluorescence signal of CPT quenched or released depending on the pH, which allowed the delivery of hybrid nanoparticles and drug release to be tracked. The above discussions indicate that AgNPs have good antibacterial properties and are good agents for in vivo drug delivery when fused with other substrates.

#### 2.3.2. Gold Nanoparticles (AuNPs)

Over the past few years, catalysis using silver, gold, and palladium has gained popularity and found extensive use in organic chemistry [53]. Although palladium is used more frequently for catalysis, specialized applications of gold owing to its anisotropic properties open up new possibilities in chemical processes.

Several characteristics of AuNPs such as their great compatibility, low toxicity, and tunable consistency make them attractive agents for drug delivery [25]. Their huge surface area and ability to interact with various substrates make them suitable for controlled drug delivery, cancer therapy, bioimaging, and diagnosis [54,55,56]. Although AuNPs are relatively safer than AgNPs, recent investigations have demonstrated that they may pass the blood–brain barrier, interact with DNA, and cause genotoxicity [57,58]. However, these risks may be reduced by selecting proper coating materials for AuNPs.

An additional advantage of AuNPs is that their surfaces can be modified more easily than those of other nanoparticles [59,60]. Using Au–thiol chemistry is the simplest technique to change the surface of AuNPs with other materials. The biomaterial to be transformed on the AuNPs can be an antibody [61] that may precisely respond to cancer cells or specific organs, and the majority are peptides [62] or proteins [63]; however, DNA strands containing thiol groups can also be modified [64,65,66]. Furthermore, amine groups can self-attach to the gold surface, allowing for the loading of numerous types of medicines [67]. When it is intended to improve biocompatibility rather than increasing target specificity, simple adsorption or electrostatic attraction may be applied for surface modification. These adherent principles can also cause drug release owing to differences in bonding strengths, and conjugated substrates could be dislodged under specific physiological conditions. For example, glutathione, which has a thiol group and is utilized as a reducing agent, is more abundant within the cell than outside, and thus, drug release is automatically facilitated when drug-loaded AuNPs are transported into the cell [68].

Rotello et al. concentrated on the engineering of the AuNP surface monolayer, emphasizing recent breakthroughs in adjusting monolayer topologies for efficient drug and biomolecule delivery [37]. Furthermore, they examined particle functionalization in organic monolayers and biomolecule coatings and explored its application in medication, DNA/RNA, protein, and small molecule delivery (Figure 1d).

### 2.4. Magnetic Nanoparticles (MNPs)

MNPs are among the most alluring forms of nanomaterials, particularly in biological applications [69]. Particularly, iron oxide-based nanoparticles are among the most commonly employed magnetic nanomaterials owing to their strong magnetic characteristics and biocompatibility. The extraordinary properties of MNPs such as high saturation field and superparamagnetism dominate the magnetic behavior of individual nanoparticles and allow their use in several biological applications [45,70].

The most popular techniques for creating MNPs include sonochemical synthesis, co-precipitation solvothermal synthesis, thermal breakdown, and microemulsion synthesis. Some benefits of chemical co-precipitation include quick and low-temperature synthesis, the non-requirement of organic reactants, and the production of desirable materials with favorable magnetic characteristics. The potential synergistic benefits of structural builders in the characteristics of the nanocatalyst, hybrid, and nanocomposite provide an alluring material for enzyme integration. Betancor et al. reported the creation of a novel stable hybrid nanocatalyst made of bioinspired silica (Si) nanoparticles that trap MNPs and horseradish peroxidase [71]. They showed that customizing the synthetic reagents and performing post-immobilization treatments significantly influenced the physical and biocatalytic characteristics of the catalysts.

For use in biomedical applications, the biocompatibility of such particles remains to be demonstrated. The biocompatibility of MNPs can be increased by coating them with biological macromolecules [72,73]. Theragnostic agents made of multilayered magnetic nanohybrid particles seem like potential options. Park et al. fabricated a novel lipid–polymer hybrid liposomal nanoplatform (HA-MNP-LPs) [38]. They created amphiphilic hyaluronic acid hexadecylamine polymers (HA-C16) (Figure 1e) that encapsulated citric acid-coated MNPs in their aqueous cores. Furthermore, the anticancer medication, docetaxel (DTX), was enclosed in the hydrophobic bilayers of liposomes. The created nanoplatform successfully facilitated targeted drug administration and controlled drug release for cancer therapy.

### 2.5. Silica Nanoparticles

Traditionally, silica nanoparticles have been created using the Stöber technique, wherein tetraethyl orthosilicate (TEOS) is used as the silica source, water and ethanol are used as solvents, and ammonia is used as a catalyst [74]. Additional silica resources employed in the fabrication of silica nanoparticles include tetramethoxysilane (TMOS), tetrakis-2-hydroxyethylorthosilicate, and trimethoxyvinylsilane.

Numerous studies on silica-based hybrids have been conducted and published and have proven that silica is an appropriate inorganic component for biological applications [75]. Silica nanoparticles have gained attention in the field of nanomedicine for increased drug absorption and protection of the medications from the harsh environment of mucosal surfaces and lumens. Owing to their relatively simple production procedures, clinical effectiveness, and potential for surface functionalization, silica nanoparticles are promising as drug carriers [76]. In addition, silica’s hydrophobicity and consistency defend susceptible molecules from degradation under certain physiological conditions such as the harsh gastrointestinal environment. Since the drug cargo in biodegradable nanocarriers (such as polymeric nanoparticles and liposomes) would leak out in the physiological environment, resulting in early drug release, solid silica nanoparticles may be preferable.

Recently, a mesoporous silica nanoparticle (MSN)-based drug delivery system has been established by employing ‘gatekeepers’ over the pore entrance (Figure 1f) [39]. In this system, the pharmaceuticals cannot be released from silica carriers unless the drug-loading system is exposed to extrinsic stimuli such as pH, redox potential, temperature, photoirradiation, or enzymes, which cause the gatekeepers to be removed. Materials with cutting-edge characteristics are becoming more and more necessary. The effectiveness of mesoporous silica in various applications is mostly attributed to its porous structure, which enables molecules to permeate into its sizable interior surface [77].

### 2.6. Polymeric Nanoparticles

Polymeric materials have been extensively investigated and employed in nanotechnology as well as in drug delivery systems. Polymeric nanoparticles (PNPs) have significant advantages including high stability and structural diversity over other nanoparticles such as liposomes [78,79]. There are two major types of polymers, natural polymers and synthetic polymers, based on their original source. Some of the most commonly used polymers are albumin, gelatin, and chitosan (natural polymers) and polylactide (PLA), poly (lactide co-glycolides) (PLGA), and polyethylene glycol (PEG) (synthetic polymers).

Dendritic polymers, often referred to as dendrimers, with a hyperbranched tree-like structure, have also gained huge scientific attention due to their precisely controlled structure and large drug-loading capacity [80]. In a recent study, Quadir et al. developed a unique gemcitabine (GEM) delivery platform with nanoarchitecture derived from dendritic polyglycerol-co-polycaprolactone (PG-co-PCL) [81]. They synthesized and tested two differently constructed block copolymers, where GEM was either covalently or non-covalently conjugated. Both of them were stable in physiological pH and able to encapsulate and release the active drug to the desired site in a pH-responsive manner. This study successfully investigated the biocompatibility and controlled drug release of developed dendritic nanostructures and also showed that the ability to modulate the release kinetics of the payload depends on the way in which GEM is connected to the drug carrier.

The availability of controlled drug release in diseased tissue is one of the critical features of nanomedicine. It is a so-called ‘smart drug delivery system’ where nanocarriers release the payload in response to internal (pH, enzymes, or redox potential) or external (magnetic field or ultrasound) stimuli (Figure 1g) [40]. PNPs, unlike other nanoparticles, have a high degree of design flexibility so that they are more likely to have stimuli-responsive elements into their structures. Although PNPs are one of the promising candidates for drug delivery, there are still challenges to be overcome such as the complexity and difficulty of scale-up synthesis. Another concern is about safety, and to overcome such a limitation, further design refinement to enhance the sensitivity of PNPs to specific conditions near the targeted site should be made.

**Table 1:** Advantages and disadvantages of biohybrid nanoparticles for drug delivery and real-time monitoring.

Nanoparticle	Advantage	Disadvantage	Refs.
Nucleic acid-based nanoparticle	Target specificity Biodegradability and biocompatibility	Potential aggregation with blood cells Adherence to the vessel wall Opsonization with plasma protein	[28,31,82]
Liposome	Structural flexibility Ease of conjugation and functionalization with contrast agents and probes Rapid cellular uptake and well-characterized cell internalization mechanism Low immunogenicity Good biocompatibility	High cost Low drug-loading efficiency Limited instability and leakage of loaded materials Rapid clearance	[41,42,83]
Silver nanoparticle	Good biocompatibility Direct cancer cell killing capability	Size-dependent cytotoxicity Potential off-target effects with little delivery to the tumor	[49,50,84]
Gold nanoparticle	Large surface area Application diversity Suitable for photodynamic therapy Ease of surface modification High stability and biocompatibility	High cost Low biodegradability Potential toxicity depends on their intrinsic characteristics	[59,60,85]
Magnetic nanoparticle	Large surface area Small size allows longer circulation and tissue penetration Controlled clustering Application diversity	Lack of colloidal stability Low biocompatibility and biodegradability In vivo toxicity	[69,72,86]
Silica nanoparticle (Mesoporous)	Large surface area High stability and biocompatibility Controllable porosity Surface reactivity and ease of functionalization Biodegradability	In vivo toxicity Low drug-loading capacity	[76,77,87]
Polymeric nanoparticle	Large drug-loading capacity Stimuli-responsive drug release Precisely controllable size Ease of fusing with other materials	Difficulty of scale-up synthesis Complex synthetic procedure Low biocompatibility	[78,88]

## 3. Biohybrid Nanoparticle-Based In Situ Drug Release Monitoring

Molecular imaging, a technique that allows imaging of the changes at various molecular levels, has been rapidly developed in recent years [89,90,91,92]. Biomolecular imaging allows us to visualize the phenomena occurring in the living state of the object. In addition, the phenomena observed through biomolecular imaging can be quantified and analyzed, providing important information for optimizing drug treatment conditions in the future [93]. The currently used molecular imaging methods in clinics which employ nanomaterials to improve their sensitivity are discussed below (Table 2).

### 3.1. Fluorescence-Based In Situ Monitoring of Drug Release

Fluorescence-based indications from potential therapeutic agents might be advantageous in determining their therapeutic efficacy, pharmacokinetic properties, and stimulation mechanism [94,95]. They can provide information on the accurate time and location of drug release. Fluorescent molecules are light-absorbing and -releasing substances that can be used as a signal in clinical diagnostics. They can attach to biomolecules such as amino acids, oligonucleotides, antibodies, and enzymes that may be traced and studied in vitro and in vivo. Efforts have been made to link fluorescent agents to drug release events in a delivery mechanism to track drug trafficking and release [96]. Furthermore, real-time information on the release process may be gathered by using non-invasive fluorescence imaging techniques.

#### 3.1.1. Förster Resonance Energy Transfer (FRET)-Based In Situ Monitoring of Drug Release

**Figure 2:**
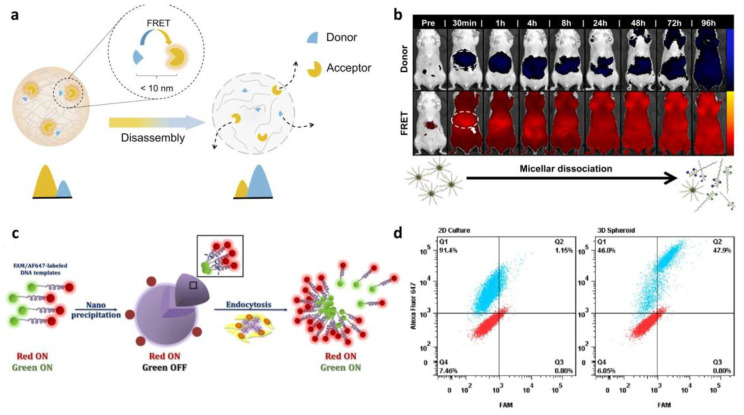
Fluorescence-based in situ monitoring of drug release. (**a**) Illustration of energy transfer between Förster resonance energy transfer (FRET) pairs. (Reprinted from Ref. [98].) (**b**) Real-time monitoring of the biodistribution of FRET-loaded micelles. (Reprinted with the permission from Ref. [99]. Copyright of 2014 Elsevier.) (**c**) Illustration of dual-labeling approach to confirm nanocomposite internalization with green fluorescence quenching effect. (**d**) Mesenchymal stem cell (MSC) uptake of the nanocomposites in the 2D and 3D (spheroid) environments. Red: control (MSC without nanocomposites); blue: MSCs treated with nanocomposites. (Reprinted with the permission from Ref. [102]. Copyright 2019 American Chemical Society.).

FRET is an analytical method for evaluating interactions between nanomedicines and biological environments [13,97]. It is a successful method to study the biological fate of NDDSs in vivo. It works based on the energy transfer between a donor fluorophore and a nearby acceptor fluorophore (Figure 2a) [98]. The distance between the FRET pair, donor and acceptor, is the key to FRET efficiency. This sensitivity to distance offers information on the nanomedicine stability in vitro and its intracellular release. Many efforts have been made to stabilize the structure of nanocarriers and track them because nanocarriers can be easily destructed or mislocalized in in vivo environments.

Co-encapsulation of the FRET pair in the core of micelles is an example of nanocarrier structure stabilization. Hammond et al. investigated the stability of FRET-loaded micelle as a drug carrier [99]. Furthermore, through the monitoring of the FRET drug-loaded micelle, real-time monitoring of drug release was successfully performed (Figure 2b). Regarding the energy transfer distance, the FRET signal significantly decreases corresponding to the release of the payload. Using FRET, drug release and the behavior of the micelle can be observed in real-time.

Although FRET has numerous advantages, several limitations remain to be overcome [100]. Re-generated FRET signal is one such limitation. It occurs when the lipophilic FRET pair is released from the nanocarrier and accumulates in intracellular cell membranes, leading to the misjudgment of drug carrier behavior or dissociation. The low signal-to-noise ratio (SNR) is another limitation of FRET associated with its imaging. Many technologies, such as acceptor photobleaching and fluorescence lifetime imaging microscopy (FLIM) FRET [101], have been developed to overcome the poor SNR observed during FRET measurements. Therefore, more improvements are required to make FRET a more reliable technology with a wide range of in vivo applications.

#### 3.1.2. Aggregation-Caused Quenching (ACQ)-Based In Situ Monitoring of Drug Release

Most fluorophores are hydrophobic due to their aromatic nature; therefore, they are likely to form aggregates in aqueous environments [12]. This is also because intermolecular hydrophobicity forces the dispersed fluorophores to form π-π stacking. As the fluorophores aggregate, the fluorescence signal is also reduced or quenched. Owing to this spontaneous reaction, ACQ has been employed for labelling and tracking nanocarriers in vivo [103].

Recently, He et al. studied the biological fate of polymeric micelles using absolute ACQ probes [104]. The fluorescent signal gradually decreased and eventually disappeared, demonstrating the stability of the synthetic micelle and the applicability of ACQ probes for bioimaging. On the other hand, Leong et al. used a dual-labeling approach to investigate the distribution of nanocomposites in a spheroid (Figure 2c) [102]. They labeled PEG and DNA with Alexa Fluor 647 and FAM, respectively, and observed decayed green fluorescence resulting from the self-quenching effect of FAM after nanocomposite formation. By monitoring the change in the fluorescence signal, the team could verify whether the internalization of the nanocomposite happened when it was cultured in 2D or 3D with mesenchymal stem cells (MSCs), providing information on its stability and location (Figure 2d).

Although ACQ-based bioimaging is a promising method for monitoring nanodrug delivery, its application is restricted to hydrophobic nanocarriers, such as nanocrystals, lipid nanoparticles, and micellar carriers, because the ACQ strategy is based on hydrophobicity [105]. Furthermore, ACQ dye may not be able to dissolve in the hydrophilic matrix of nanocarriers, leading to failure of nanocarrier illumination. By optimizing the structure of the currently available dyes and discovering new ACQ dyes, the ACQ-based bioimaging strategy can be made more accurate and reliable for nanodrug carrier tracking [106].

### 3.2. Surface-Enhanced Raman Spectroscopy (SERS)-Based In Situ Monitoring of Drug Release

Unlike the aforementioned fluorescence-based monitoring methods, SERS is a biomolecular sensing and imaging method that does not require additional dyes [107]. Raman spectroscopy could give us molecule-specific information by detecting photons scattering from their vibrational energy level; however, its application is limited by the inherent small Raman scattering cross-section, which results in poor sensitivity. Thus, SERS was developed by positioning analytes adjacent to metal nanostructures, resulting in a significantly enhanced Raman signal and increased single-molecule sensitivity [15]. As NDDSs have been attracting great attention, further investigation of their mechanism and effects on living cells is required, and SERS is expected to perform highly sensitive and time-dependent monitoring of drug delivery in a living subject [108].

**Figure 3:**
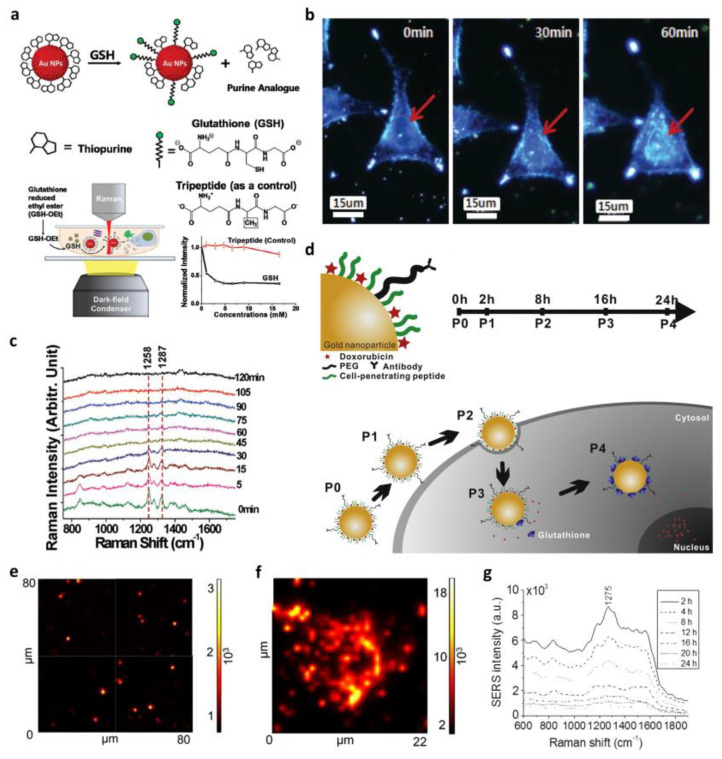
Surface-enhanced Raman spectroscopy (SERS)-based in situ monitoring of drug release. (**a**) GSH-mediated drug release monitoring in live cells, investigated by monitoring the decrease in SERS intensity. (**b**) Time-lapse cell images (0 min, 30 min, and 60 min) and (**c**) Raman spectra. The peak shifting from 1258 cm^−1^ to 1287 cm^−1^ indicates in situ drug release. (Adapted with permission from Ref. [109]. Copyright 2012 American Chemical Society.) (**d**) Schematic diagram of biohybrid nanoparticle synthesis and time-dependent monitoring of its intracellular delivery and drug release; SERS map images of biohybrid nanoparticle treated with (**e**) SH-SY5Y (control) and (**f**) SK-BR-3 cells (target cell), respectively, to investigate target specificity. (**g**) SERS spectra to observe time-dependent drug release from biohybrid nanoparticles. (Adapted with permission from Ref. [110]. Copyright 2015 Elsevier.).

Recent studies have employed SERS for label-free in situ monitoring of drug release. Ock et al. demonstrated the application of SERS for in vitro and in vivo label-free monitoring of real-time drug release (Figure 3a–c) [109]. They successfully confirmed extracellular and intracellular glutathione (GSH)-mediated drug release from AuNP surfaces by imaging a live cell and observing the decrease in SERS intensities in real time. Meanwhile, Choi et al. proposed an in situ label-free monitoring and controlled intracellular drug delivery system using biohybrid nanoparticles based on SERS for the first time [110]. For more specific cell targeting and a higher uptake, the hybrid nanoparticle was made using AuNPs, a cell-penetrating peptide (CPP), and a target cell antibody (Figure 3d). The distribution of the nanoparticles inside cells and their target specificity were studied by comparing SERS map images obtained from different cell lines (Figure 3e,f). Furthermore, time-dependent drug release inside the target cells, SK-BR-3, was successfully observed through increased SERS intensity (Figure 3g). They showed the capacity of biohybrid nanoparticles to act as aromatic anticancer drugs and the efficiency of SERS as a spectroscopic biosensor for in situ drug release.

Although SERS is a reliable technique for biomolecular analysis, some challenges remain to be addressed [111]. One of the controversial issues is the cytotoxicity of plasmonic nanoparticles. Future investigations on SERS should focus on developing coating materials to alleviate toxicity and improve target specificity. The SERS-based sensing method can expand the research area of biological science with a better understanding of the mechanisms of nanoparticles in biological systems.

### 3.3. Magnetic Resonance Imaging (MRI)-Based In Situ Monitoring of Drug Release

One of the most popular methods for monitoring medical conditions is MRI, which is non-invasive and repeatable over time [112]. It is also a promising technique for monitoring the specific targeting and biodistribution of nanodrugs. MRI is based on the *T*_1_ or *T*_2_ relaxation times of protons from different biomolecular structures to create imaging contrast. *T*_1_, the spin–lattice relaxation time, refers to the time taken by an excited proton to revert to its original state, whereas *T*_2_, the spin–spin relaxation time, describes how quickly the magnetic resonance (MR) signal fades after excitation. The MR image contrast depends on whether *T*_1_ or *T*_2_ is weighted. For instance, as fat loses transverse magnetization more rapidly than water, it will appear brighter in a *T*_1_-weighted image than in a *T*_2_-weighted one. Thus, MRI monitoring is suitable for the monitoring drug release of nanocarriers and its efficacy is based on the contrast-generating principle, which offers different signals when a drug is in or out of the nanocarrier [113].

**Figure 4:**
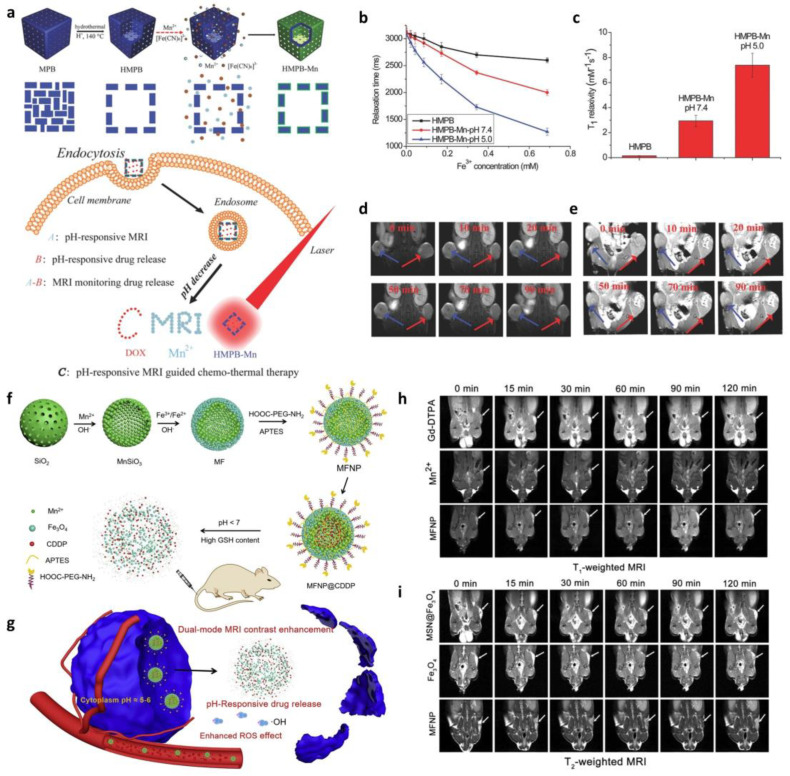
MRI-based in situ monitoring of drug release. (**a**) Illustration of synthetic procedure and intracellular MRI monitoring of Mn-containing nanoparticles. (**b**) Longitudinal relaxation time and (**c**) *T*_1_ relaxivity of HMPB and HMPB-Mn at different pH conditions. (**d**) Representative dynamic contrast-enhanced MRI of a tumor site treated with HMPB and (**e**) HMPB-Mn. (Reproduced with permission from Ref. [114]. Copyright 2015 WILEY-VCH Verlag GmbH & Co. KGaA, Weinheim.) (**f**) Schematic diagram of the synthetic procedure for magnetic ferrite nanoparticles (MFNPs) and (**g**) its abilities for cancer therapy and diagnosis. Time-dependent *T*_1_-weighted (**h**) and *T*_2_-weighted (**i**) MR images of mice injected with different NPs. (Adapted with permission from Ref. [116]. Copyright 2019 Elsevier.).

Considerable efforts have been made to develop contrast agents for enhancing imaging sensitivity and resolution. Figure 4a shows the synthetic process of multifunctional theranostics [114]. Hollow mesoporous Prussian blue nanoparticles (HMPBs) were coated inside and outside with a manganese (a typical contrast agent)-containing Prussian blue analogue (MnPBA), forming the core–shell hollow-structured HMPB-Mn. When the pH-sensitive HMPB-Mn reached the target tumor site, it released Mn^2+^ ions which made the HMPB-Mn become a super sensitive *T*_1_-weighted contrast agent with higher relaxivity than naked HMPB without Mn coating (Figure 4b,c). Moreover, the real-time monitoring of drug release through MRI was enabled because the Dox loaded in HMPB-Mn was also released in a pH-triggering manner (Figure 4d,e). Although contrast agents have played a great role in MRI imaging, the dynamic endogenous conditions often make it difficult to accurately interpret the MRI signals. To overcome such problems, dual-mode contrast agents are gaining academic attention [115]. For example, Sun et al. demonstrated a biodegradable nanoplatform for dual-mode MRI-guided combinatorial cancer therapy (Figure 4f,g) [116]. They blocked the pores on manganese silicate (MnSiO_3_) by conjugating iron oxide nanoparticles onto the surface of it to prevent the possible leakage of drugs loaded inside. The nanoplatform is destructed in the tumor microenvironment (TME), where GSH concentration is high and pH is under 7, resulting in drug release due to Fe_3_O_4_ disassociation from the nanoplatform. The released Fe_3_O_4_ and Mn^2+^ then help enhance the MRI contrast by reducing the intervention in *T*_1_ or *T*_2_ contrast abilities (Figure 4h,i). The biodegradability and drug delivery efficacy of the nanoplatform was successfully investigated in vitro and in vivo, suggesting that the nanoplatform is a promising material for dual-mode MRI and cancer therapy.

**Table 2:** Advantages and disadvantages of different techniques and their contrast agents used for in vivo monitoring.

Methods	Contrast Agents	Advantage	Disadvantage	Refs.
Fluorescence-based	Various fluorescence-conjugated biomolecules Fluorophore	Non-invasive Radiation-free Available to combine with other imaging methods	Dye cytotoxicity Limited tissue penetration depth	[13,100,105,117]
SERS	Raman reporter-conjugated gold nanoparticle	Ease of sample preparation Narrow peak width allowing multitarget detection	Low intensity and poor reproducibility Difficulty of quantitative analysis	[107,111]
MRI	Paramagnetic ions (Gadolinium, Manganese, and Iron)	Non-invasive Highly spatial and temporal resolutions Possible to use external magnetic field to manipulate drug carriers and/or cells	Relatively high toxicity of nanoparticles Possibility of signal affected by contrast agents when using superparamagnetic particles Inability to distinguish live cells from dead ones	[112,115]
Ultrasound	Microbubbles	Radiation-free Can detect single cells Relatively inexpensive Allows imaging of soft tissues	Low resolution Restricted to specific parts of body Difficulty of quantification Contrast agent can transfer to non-target cells	[118,119]

SERS, surface-enhanced Raman spectroscopy; MRI, magnetic resonance imaging.

## 4. Fate of Biohybrid Nanoparticles

Monitoring drug release in vitro and in vivo is critical for diseased tissues, improving therapeutic efficiency, and understanding the pharmacokinetic features of NDDSs [16,90,120]. In this regard, developing strategies for real-time monitoring of the fate of nanodrugs in biological systems is essential.

Nanocarriers are usually designed with surface modifications such as targeting ligands or coating materials for enhanced target specificity, stability, and delivery efficiency [1]. Drug distribution to the desired tissue often fails due to pre-degradation or loss of the nanocarrier coating, leading to leakage of payloads [121]. Meanwhile, mislocated or accumulated nanocarriers sometimes induce intracellular toxicity or phagocytic reaction [122]. Thus, nanocarriers must be both stable and degradable in biological systems. However, studies on the stability and degradation of nanocarriers in biological contexts have been limited in the past. Here, we will overview the recent studies applying monitoring methods to track nanocarrier fate and nanocarrier disintegration-based drug release.

### 4.1. Tracking-Based In Vivo Drug Delivery Monitoring

**Figure 5:**
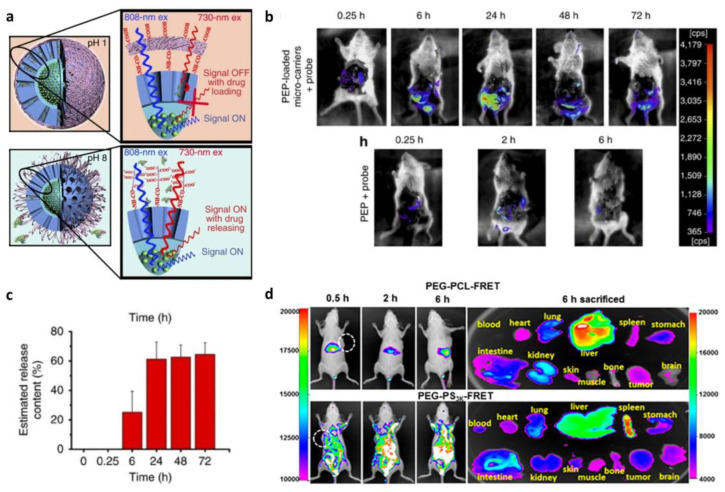
Nanodrug tracking. (**a**) Illustration of microcarrier fate tracking. (**b**) In vivo monitoring of drug release via NIR-II bioimaging and (**c**) time-dependent quantitative analysis of drug release. (Adapted from Ref. [123].) (**d**) In vivo behavior of two different polymeric micelles (PEG-PCL and PEG-PS) based on FRET analysis. (Adapted with permission from Ref. [125]. Copyright 2018 American Chemical Society.).

Recently, Wang et al. reported a novel microcarrier that can be delivered orally and can withstand the gastrointestinal environment (Figure 5a) [123]. The microcarrier core consisted of a lanthanide-based downconversion nanoparticle (DCNP), which can emit in the second near-infrared (NIR-II) window. Fluorescence imaging in NIR windows, NIR-I (700–900 nm) and NIR-II (1000–1700 nm), can overcome the limitations that other fluorescent-based imaging techniques often face, such as autofluorescence, self-quenching, and failure of tissue penetration [117]. It facilitates deeper tissue penetration depths and higher resolution with less photon scattering and minimized autofluorescence compared to other fluorophores. However, there are not many NIR-II emitting probes available in biomedical fields due to several obstacles including toxicity, low biocompatibility, and solubility. In this point of view, it is important that this study designed a NIR-II traceable microcarrier with higher stability and biocompatibility. Furthermore, they fabricated an absorption competition-induced emission (ACIE) bioimaging system for simultaneous in vivo microcarrier fate tracking and semi-quantitative monitoring of drug-release percentage (Figure 5b,c). Using this bioimaging technique, they successfully investigated the retention behavior of the microcarrier depending on its size and confirmed its low health risk by histopathological examination after administering the microcarrier for a week.

Polymer micelle, one of the most commonly used nanocarriers, has already entered clinical trials; however, its therapeutic efficiency is unexpectedly low owing to its fast disassociation in vivo [124]. To monitor the behavior of differently designed polymer micelles in the bloodstream, Sun et al. synthesized polyethylene glycol-*block*-poly (ε-caprolactone) (PEG-PCL) and poly(ethylene oxide)-*block*-polystyrene (PEG-PS) and investigated their delivery efficacy [125]. They conjugated FRET dyes to the hydrophobic ends of the polymers to avoid dye release and track the post-injection behavior of the micelles in vivo and ex vivo via fluorescence imaging (Figure 5d). Both of the micelles were rapidly disassociated into unimers after IV injection, and then only PEG-PCLs were accumulated mostly in the liver by macrophages and Kupffer cells, while PEG-PSs were spread over the whole body. This study showed the clearance mechanism of polymer micelles after injection and once again addressed the importance of the stability of micelles for effective drug delivery.

### 4.2. Degradation-Based In Vivo Drug Delivery Monitoring

**Figure 6:**
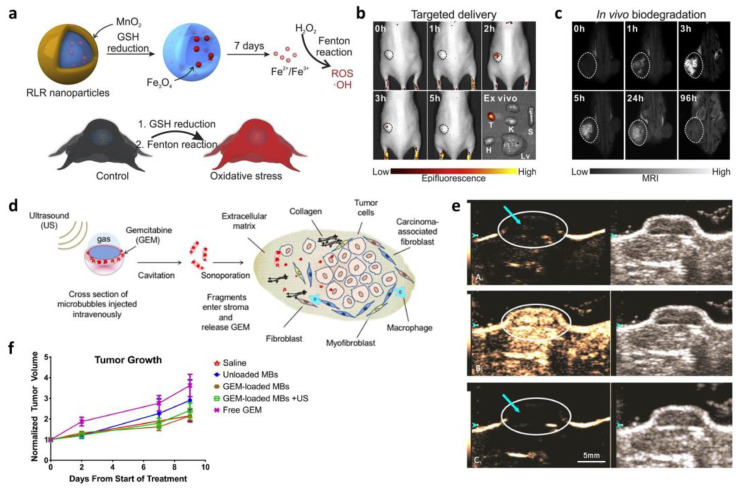
(**a**) Schematic diagram depicting the mechanism of a redox and light-responsive (RLR) nanoparticle-associated synergistic chemodynamic therapy (CDT). (**b**) Fluorescence image of time-dependent target specificity monitoring. (**c**) Confirmation of in vivo biodegradation of the nanoparticles via MR imaging. (Adapted with the permission from Ref. [127]. Copyright 2019 Elsevier.) (**d**) Schematic diagram of microbubble drug delivery in an ultrasound-triggered manner. (**e**) Ultrasound-based tracking of drug carrier and drug release at the tumor site. (**f**) Treatment of a tumor using nanocarriers. (Reprinted with the permission from Ref. [128]. Copyright 2021 Elsevier.).

Chemodynamic therapy (CDT) is a cancer treatment method that induces ferroptotic pathways in cancer cells by converting H_2_O_2_ into •OH, improving the specificity of treatment compared to traditional methods [126]. However, the low conversion rate of H_2_O_2_ into •OH limits its clinical application; therefore, many studies have reported novel methods for improving the catalytic efficiency of CDT-based cancer therapy. Recently, Lee et al. developed a redox and light-responsive (RLR) nanoparticle that can be programmatically degraded and can accelerate reactive oxygen species (ROS) generation for CDT-induced cancer cell killing (Figure 6a) [127]. It has iron oxide nanoparticles in the core, which is embedded in a carbon framework covered with an MnO_3_ shell which will be degenerated inside cancer cells and release the MR imaging agent Mn^2+^. They injected a fluorescein isothiocyanate (FITC)-labeled nanoparticle to investigate cancer cell specificity and confirmed a significant increase in fluorescence signals at the tumor site (Figure 6b). Its degradability was also examined by MR imaging in vivo and indicated selective accumulation and degeneration at the cancer site, which are crucial considerations for their clinical application (Figure 6c).

Ultrasound imaging is another non-invasive imaging technique for drug release monitoring apart from fluorescence imaging, SERS, and MRI [118]. It has attractive characteristics, such as real-time monitoring, high accessibility, and superior safety. Furthermore, the therapeutic potential of contrast agent microbubbles (MBs) has been acknowledged in recent studies [119]. For instance, Wheatley et al. demonstrated drug-encapsulated MB for ultrasound imaging and cancer therapy (Figure 6d) [128]. Gemcitabine (GEM), a commonly used drug for pancreatic cancer treatment, was entrapped in polymer MBs and protected from degradation in the plasma. The difference in tumoral contrasts enhanced by the MB was clearly observed in Figure 6e, suggesting their degeneration inside the target tumor before and after destructive pulses. The target specificity and degradability of GEM-MBs were successfully investigated; however, no significant reduction in tumor growth was observed across any of the treatment groups (Figure 6f). This result indicates that although the delivery system was tolerable and specific, the amount of GEM loaded in MBs was not high enough to cause therapeutic effects. Therefore, with further investigation on the drug-loading capacity, the MB-associated delivery platform is expected to be applied for pancreatic cancer therapy owing to its target specificity that enables the safeguarding of healthy tissues.

## 5. Conclusions and Future Perspectives

Nanoparticle-based drug delivery systems have numerous advantages, including high specificity to target sites, controllable drug release, and enhanced therapeutic efficacy compared to traditional drug delivery systems. However, the possibilities of aggregation and immunoreaction against nanodrug carriers often limit their clinical application. To overcome such obstacles, many studies have reported innovative nanomaterial-based drug delivery platforms and highlighted the importance of in vivo monitoring methods.

Bioimaging methods for tracking the destiny of nanocarriers have been discussed in this review, with an emphasis on nanocarrier degradation and drug release mechanisms. However, encapsulated medications often fail to reach target sites when the nanocarrier is destructed too soon or has poor specificity. On the other hand, the toxicity of the nanomaterials in vivo is another issue that also needs to be addressed. This is not only because the core of the nanodrug carrier itself is less biocompatible, but it is also because mislocated nanodrugs can harm healthy tissue or be accumulated in the body. The key solution to these issues is to develop appropriate design strategies of nanocarriers and track them using various imaging methods to understand how they react inside our bodies. With more detailed and delicate construction of nanodrug carriers, the current status of nanoparticle-based therapy will be boosted and evaluated to perform efficient and safe treatment. In this regard, this review has focused on the basis of nanoparticles and their recent applications in drug delivery and bioimaging techniques.

In conclusion, future research on nanoparticle-based drug delivery should address their biological effects on living subjects. Furthermore, the biodistribution of the physicochemical properties of the developed nanomaterials and the interactions between biological systems and nanocarriers must be examined in vitro and in vivo using appropriate monitoring methods. Additionally, the surface modification or functionalization of nanocarriers for improving the therapeutic efficacy, stability, and biocompatibility of nanoparticle-based drug delivery systems is essential.
